# Geographical and taxonomic patterns in aerobic traits of marine ectotherms

**DOI:** 10.1098/rstb.2022.0487

**Published:** 2024-02-26

**Authors:** Justin L. Penn, Curtis Deutsch

**Affiliations:** ^1^ Department of Geosciences, Princeton University, Princeton 08544, NJ, USA; ^2^ High Meadows Environmental Institute, Princeton University, Princeton 08544, NJ, USA

**Keywords:** hypoxia tolerance, metabolism, biogeography, ecophysiology, marine ectotherms, climate change

## Abstract

The metabolism and hypoxia tolerance of marine ectotherms play key roles in limiting species geographical ranges, but underlying traits have only been directly measured for a small fraction of biodiversity. Here we diagnose and analyse spatial and phylogenetic patterns in hypoxia tolerance and its temperature sensitivity at ecologically active metabolic rates, by combining a model of organismal oxygen (O_2_) balance with global climate and biogeographic data for approximately 25 000 animal species from 13 phyla. Large-scale spatial trait patterns reveal that active hypoxia tolerance is greater and less temperature sensitive among tropical species compared to polar ones, consistent with sparse experimental data. Species energetic demands for activity vary less with temperature than resting costs, an inference confirmed by available rate measurements. Across the tree of life, closely related species share similar hypoxia traits, indicating that evolutionary history shapes physiological tolerances to O_2_ and temperature. Trait frequencies are highly conserved across phyla, suggesting the breadth of global aerobic conditions selects for convergent trait diversity. Our results support aerobic limitation as a constraint on marine habitat distributions and their responses to climate change and highlight the under-sampling of aerobic traits among species living in the ocean's tropical and polar oxythermal extremes.

This article is part of the theme issue ‘The evolutionary significance of variation in metabolic rates’.

## 1. Introduction

The distribution of species across space and time is strongly influenced by physiological sensitivities to environmental conditions. In the ocean, oxygen (O_2_) is increasingly recognized as a constraint on thermally and metabolically available habitat [1–4], even outside of O_2_ minimum zones, and in waters with O_2_ well above the traditional definition of hypoxic (less than 60 µM) [5,6]. Species sensitivity to low O_2_ levels varies with temperature (T) and body size (B), owing to the thermal and allometric dependence of metabolism and physiological O_2_ supply [7–9]. Limitation of growth by O_2_ remains a leading hypothesis for the Temperature Size Rule [10–14].

Species hypoxia tolerance traits, such as the ratio of organismal O_2_ supply and metabolic demand rates and their dependence on temperature, body size, and activity level can be used to map available aerobic habitat in the ocean [4,9,15–19]. Aerobic habitat barriers predicted from these traits and observed climate fields commonly align with the geographical range boundaries of species across ocean basins, latitudes and depths. Measured traits can thus help predict how the lowest O_2_ level inhabited by species varies as a function of ocean temperature. They also mediate critical responses to climate change, including present and historical habitat exclusion [16,18,20], intra-specific body size variations [14], and marine mass extinction [21]. These traits are also expected to be strong determinants of future extinction risk from anthropogenic climate warming under ongoing O_2_ loss [22,23]. Quantifying how they vary across the ocean and the tree of life is key for a mechanistic understanding of biogeographic, taxonomic and evolutionary responses to climate.

**Figure 1.**
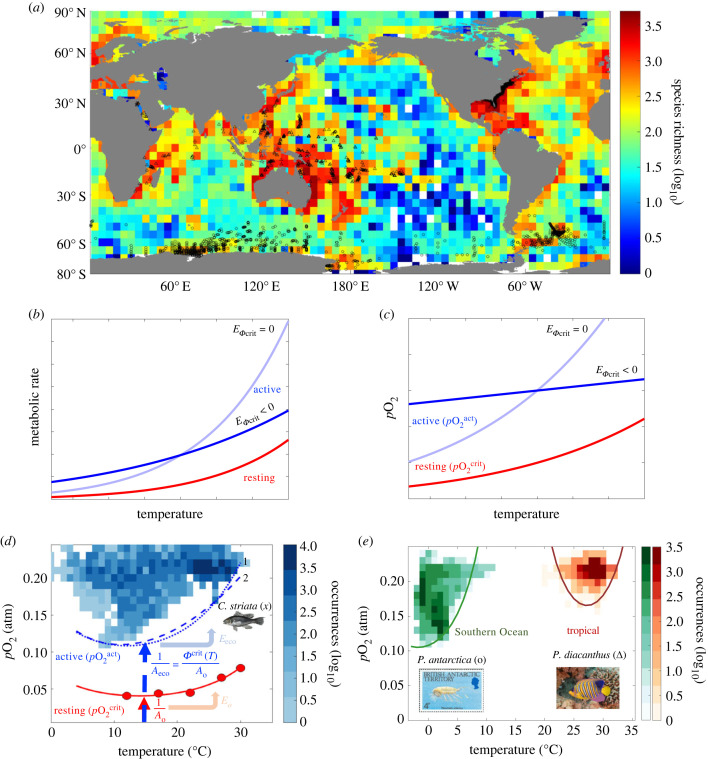
Temperature-dependent hypoxia traits estimated from biogeographic observations. Traits are diagnosed from paired geospatial occurrences, and temperature and partial pressure of oxygen (T-pO_2_) observations for globally diverse species (*a*; species richness). (*b,c*) Active and resting metabolic rates increase with temperature (*b*), giving rise to temperature-dependent lower pO_2_ thresholds required to maintain metabolism, ${\mathrm{pO}}_{2}^{\mathrm{act}}$ and ${\mathrm{pO}}_{2}^{\mathrm{crit}}$, respectively (*c*). If the ratio of active to resting metabolic rate is constant versus temperature $\left(E_{\Phi \mathrm{crit}}=0\right)$, ${\mathrm{pO}}_{2}^{\mathrm{act}}$ and ${\mathrm{pO}}_{2}^{\mathrm{crit}}$ vary similarly with temperature (compare light blue and red lines). By contrast, if activity is less temperature sensitive than resting costs $\left(E_{\Phi \mathrm{crit}}<0\right)$, ${\mathrm{pO}}_{2}^{\mathrm{act}}$ varies less with temperature than ${\mathrm{pO}}_{2}^{\mathrm{crit}}$ (compare dark blue and red lines). (*d,e*) Species occurrences in T-pO_2_ state-space (shading) reveal how the minimum pO_2_ inhabited by a species in the ocean $(pO_{2}^{\mathrm{act}})$ varies with T. This active T-dependence (*E*_eco_) reflects both the resting hypoxia tolerance (*E*_o_) measured by ${\mathrm{pO}}_{2}^{\mathrm{crit}}$ (red points in *d*) and the ratio of active to resting metabolic costs $\left(E_{\Phi \mathrm{crit}}\right)$. Active hypoxia tolerances (*A*_eco_) at the reference T (15°C) are reduced relative to the resting state (*A*_o_) by the ratio of active to resting metabolic rates $\left(\Phi_{\mathrm{crit}}\right)$. T-dependences can vary across a species temperature range owing to the multi-step nature of O_2_ supply, as captured by including multiple Arrhenius functions (dashed line 2 in *d*) [25], or by allowing *E*_o_ and *E*_eco_ to be a linear function of T, with slope $\left(\frac{dE}{dT}\right)$ (dotted line 1 in *d*). T-pO_2_ occurrences (*d,e*) and geographical distributions (symbols in *a*) are shown for: (*d*) western Atlantic black sea bass (*Centropristis striata, x*) [26], (*e*) the Southern Ocean copepod (*Paraeuchaeta antarctica*, o), and tropical Indo-Pacific royal angelfish (*Pygoplites diacanthus,* Δ). Traits are estimated by fitting a model of O_2_ supply to demand (electronic supplementary material, equation S1, S8; lines) to experimental ${\mathrm{pO}}_{2}^{\mathrm{crit}}$ data and inhabited lower pO_2_ levels. Illustrations in (*d*) and (*e*) are from NOAA FishWatch (https://en.wikipedia.org/wiki/Black_sea_bass#/media/File:Centropristis_striata.png) and WoRMS (https://www.marinespecies.org/aphia.php?p=image&pic=46884&tid=344974), respectively, and photograph in (*e*) is from D. Delso (https://en.wikipedia.org/wiki/Royal_angelfish#/media/File:Pez_%C3%A1ngel_real_(Pygoplites_diacanthus),_parque_nacional_Ras_Muhammad,_Egipto,_2022-03-26,_DD_155.jpg).

While the traits governing temperature-dependent hypoxia tolerance have been estimated for dozens of marine species from direct respirometry experiments [9], they still represent a small fraction of known marine species [24], with the potential for regional, phylogenetic, and experimental biases. Hypoxia traits estimated from biogeographic observations reflect those directly measured in laboratory experiments, modified by ecological factors, including differences between resting versus active metabolism. Here we pair a model of organismal O_2_ balance with global biogeographic data and climatological measurements of temperature and O_2_ to diagnose temperature-dependent hypoxia traits from a database of 24 852 marine animal species (figure 1*a*), and analyse trait correlations, spatial patterns across the ocean and phylogenetic signals across the tree of life.

## 2. Methods

The minimum partial pressure of oxygen (pO_2_) required to maintain metabolism is a measure of physiological vulnerability to hypoxia (figure 1*b,c*). A species minimum pO_2_ threshold can vary with temperature and increases with activity level relative to the resting state, where it is defined as *V*_h_ (in atm) at an arbitrary reference temperature (see the electronic supplementary material, table S1 for definitions of mathematical symbols). The ecological activity of a viable population in nature is vulnerable to O_2_ limitation at a higher pO_2_ level, *V*_h_*Φ_crit_, where *Φ*_crit_ is the ratio of sustained to resting metabolic rate and falls between 1 (i.e. no active costs) and the ratio of maximum to resting metabolic rate, defined by the factorial aerobic scope (FAS) and commonly measured in animal physiology [9,27,28]. This active hypoxia vulnerability of a species is inversely related to its active hypoxia tolerance, which we denote *A*_eco_ (1/atm), such that *A*_eco_ = 1/(*V*_h_*Φ_crit_), following analogous definitions for resting hypoxia tolerance and vulnerability at minimum metabolic rate, i.e. *A*_o_ = 1/*V*_h_ [9] (figure 1*d*). Thus, for an ecologically active population, the minimum pO_2_ in a metabolically viable environment (pO_2_^act^) can be described by:

2.1 $${\mathrm{pO}}_{2}^{\mathrm{act}}=\frac{1}{A_{\mathrm{eco}}}f(E_{\mathrm{eco}},T),$$

where *f(E*_eco_, *T*) is the variation in active hypoxia tolerance as a function of temperature and the species-specific thermal sensitivity, *E*_eco_. Variations in hypoxia tolerance owing to body size are relatively small [14,29], and we therefore neglect the allometric term here for simplicity (but see the electronic supplementary material).

Hypoxia tolerance is commonly observed to vary nonlinearly with temperature (figure 1*d*) and recent experimental data indicate it can even be non-monotonic, reaching a maximum tolerance (minimum vulnerability) at a temperature optimum [16,25,30]. The functional form of *f*(*E*_eco_, *T*) must thus accommodate a wide range of temperature dependencies. The exponential Arrhenius function of temperature provides a natural starting point, because it describes many chemical and biological rates, including metabolism (O_2_ demand) and diffusion (O_2_ supply), as well as external ventilation and internal circulation over a limited temperature range:

2.2 $$f(E,T)=\exp\left(\frac{-E}{k_{B}}\left[\frac{1}{T}-\frac{1}{T_{\mathrm{ref}}}\right]\right),$$

where *E* is an activation energy (in electron volts, eV) and represents the thermal sensitivity of the rate of any process, or the ratio of such rates. The function, *f*, increases with temperature when *E* > 0 and decreases with temperature when *E* < 0. For resting hypoxia tolerance, which is defined by the ratio of O_2_ supply to resting metabolic demand (electronic supplementary material) [4,9], its temperature sensitivity, *E*_o_, is the difference between the effective activation energies for metabolic demand (*E*_d_) and O_2_ supply, *E*_s_ (*E*_o_ = *E*_d_−*E*_s_) [9]. This formulation captures much of the thermal variation in resting hypoxia tolerance from laboratory measurements.

To represent active hypoxia tolerance across the full range of ocean temperatures, the temperature sensitivity of the Arrhenius equation requires two distinct modifications. First, metabolic rates needed to fuel ecological activity will elevate hypoxia vulnerability by the ratio of sustained to resting metabolic rates, *Φ*_crit_, which in principle may also vary with temperature. We assume that this ratio also follows an Arrhenius function of temperature, with a distinct temperature sensitivity, denoted *E_Φ_*_crit_, a species trait that may differ from that of resting metabolism (*E*_d_). Including this additional component of ecologically critical energy demand, the net temperature dependence of active hypoxia tolerance, which we denote *E*_eco_, becomes a sum *E*_eco_ = *E*_o_ + *E_Φ_*_crit_ = *E*_d_ - *E*_s_ + *E_Φ_*_crit_. To the degree that the ratio of energetic expenditure on activity versus maintenance is constant across a species temperature range, *E_Φ_*_crit_ will equal 0 and the *E*_eco_ diagnosed from biogeographic data should be equal to that of resting hypoxia tolerance derived from laboratory experiments (figure 1*b,c*). By contrast, if the energy spent on ecological activity increases less with temperature than resting costs, the ratio of active to resting energy will decline (*E_Φ_*_crit_ < 0), and *E*_eco_ should be lower than its physiological counterpart, *E*_o_. Thus, the comparison of biogeographically estimated *E*_eco_ with laboratory derived *E*_o_ can reveal new information about how temperature influences species allocation of energy to activity relative to maintenance needs.

A second modification of the simple Arrhenius function to describe hypoxia tolerance arises from the multiple steps in the O_2_ supply chain, which can have distinct activation energies (*E*_s_ values). This leads to an effective *E*_o_ that itself can vary across a species temperature range (*dashed blue line 2, figure 1d*) [25]. The complexity of these physiological processes can be represented in a dynamic organismal model of metabolism and O_2_ transport that reproduces observed thermal optima in hypoxia tolerance [25]. The diversity of resulting hypoxia tolerance curves can be simply parameterized by allowing *E*_o_ to be a linear function of *T*, with slope $\left(\frac{dE}{dT}\right)$ (electronic supplementary material, equation S4), which captures the multistep nature of O_2_ supply with minimal additional parameters (dotted blue line 1, figure 1*d*).

We estimated the ecophysiological traits (*A*_eco_ and *E*_eco_, and $\frac{dE}{dT}$) that define the minimum pO_2_ to sustain active aerobic metabolism by combining a model of organismal O_2_ supply to demand rates, the metabolic index (*Φ*; electronic supplementary material, equation S1, S8), with species geospatial occurrence data and long-term mean observed fields of ocean temperature and O_2_ (electronic supplementary material) [31–33]. Geospatial occurrences (*n* = 20 441 987) were downloaded from the Ocean Biodiversity Information System (OBIS; https://obis.org/) for 25 090 marine ectothermic animal species (figure 1*a*; electronic supplementary material, table S2) and paired to gridded hydrographic data (1° × 1°) from monthly climatological fields from the World Ocean Atlas. Traits were diagnosed by projecting species geospatial occurrences onto the temperature and partial pressure of oxygen (T-pO_2_) state-space that they inhabit, using a standard statistical categorization metric, the F1-score (electronic supplementary material, equation S10), to maximize the skill of *Φ* in predicting the observed lower pO_2_ threshold (pO_2_^act^; electronic supplementary material, equation S9) above which occurrences are common and below which they are rare, as a function of ocean temperature (electronic supplementary material, figure S1) [18]. In total, this procedure yields trait estimates for 24 852 species from 13 marine animal phyla and 51 classes with global coverage, including cosmopolitan species, and those endemic to the Northern and Southern Hemisphere's high and mid-latitude waters, and the tropics (figure 1*a*; electronic supplementary material, figure S2).

## 3. Results

**Figure 2.**
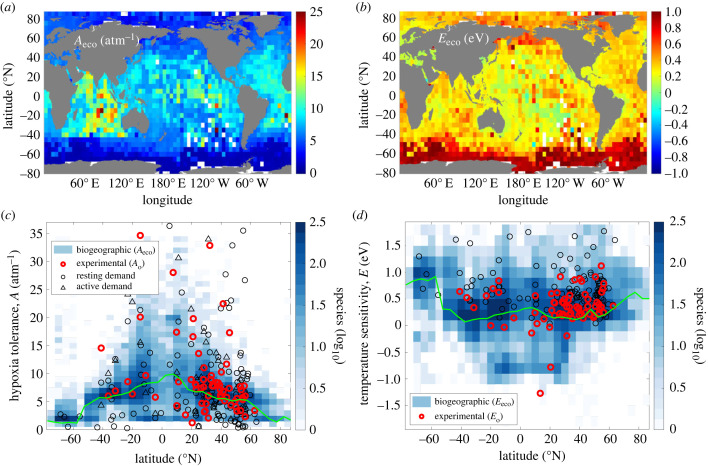
Geographical variation of active hypoxia tolerance and its temperature sensitivity and underlying metabolic traits. (*a,b*) Maps of interspecies median active hypoxia tolerance (*A*_eco_) and its temperature sensitivity (*E*_eco_) reveal large-scale gradients in traits, including a polar to tropical increase in *A*_eco_ and decrease in *E*_eco_. (*c,d*) Diagnosed traits (colours; log_10_ number of species) vary with latitude similar to available resting traits from experimental data (red points) and can be understood as arising from spatial variations in resting and active metabolic O_2_ demand (black circles and triangles, respectively in *c*) and their temperature dependences (black circles in (*d*) show resting *E*_d_), with the potential for contributions from organismal O_2_ supply. In (*c*), metabolic contributions to *A*_eco_ are predicted from the ratio O_2_ supply to demand rates (*A*_eco_ = *α*_S_/(*α*_D_*Φ_crit_)), using latitudinal variations in either measured resting metabolism (*α*_D_) (circles) or active metabolism (*α*_D_*Φ_crit_) (triangles) while holding other factors, like the efficacy of O_2_ supply (*α*_S_), at median experimental values (*Φ*_crit_ data from [9]). Median diagnosed trait values versus latitude are shown as solid green lines. In (*c,d*), species traits are plotted at their corresponding median latitude. In (*c*), resting hypoxia tolerances, *A*_o_ (red), are normalized by a constant *Φ*_crit_ of 3.

Across species, active hypoxia tolerance and its temperature sensitivity exhibit large-scale geographical patterns (figure 2). Active hypoxia tolerances increase systematically from the poles to equator, with lowest values throughout the Southern Ocean and a broad maximum across the low latitudes, reflecting the need for species to support active aerobic metabolism at warmer water temperatures and lower ambient pO_2_ in the tropics (figure 2*a,c*). By contrast, the temperature sensitivity of active hypoxia tolerance generally decreases from the poles to (sub)tropics with a weaker secondary peak at the equator, corresponding to the equatorial Pacific upwelling zone (figure 2*b,d*). This latitudinal decline in *E*_eco_ indicates the common pattern of a decrease in hypoxia tolerance from warming becomes weakened. Peak *E*_eco_ values are found in Southern Ocean species, and to a lesser extent those from the north Pacific and Arctic (figure 2*b,d*), as this trait provides habitat in cold waters (e.g. *Paraeuchaeta antarctica* in figure 1*a,e*). Species exhibiting negative *E*_eco_, which indicates that hypoxia tolerance instead increases with temperature, are most frequently found in the tropics and subtropics (e.g. *Pygoplites diacanthus* in figure 1*a,e*), but even in these regions, median species *E*_eco_ remains positive. Diagnosed differences in the distributions of *A*_eco_ and *E*_eco_ between the low (less than 30^o^) and high latitudes (greater than 60°) are statistically significant (two-sample Kolmogorov-Smirnov test, *p* < 10^-3^) (electronic supplementary material, figure S3). Measured resting hypoxia tolerance (*A*_o_) is also higher in the tropics where negative *E*_o_ values are found (figure 2*c,d*), indicating that the geographical structure of resting traits from laboratory respirometry experiments is consistent with patterns in active traits.

Variability in species active hypoxia tolerance also increases from the poles to equator, corresponding to the stronger variance of T and O_2_ associated with their vertical gradients (figure 2*c*). While the central tendency and upper deciles of *A*_eco_ are higher in the tropics, these regions also host many species with low hypoxia tolerance. Some species endemic to the tropics or high latitudes can appear to require pO_2_ above atmospheric levels (i.e. *A*_eco_ < 4.8 atm^−1^), because hypoxia tolerance is defined at a common reference temperature that lies outside their native range (figure 1*e*). Low hypoxia tolerance can also reflect species living in supersaturated O_2_ conditions found in surface waters or in the abyssal sea, where pO_2_ can exceed that of the surface atmosphere (approx. 0.21 atm).

Spatial variations in active hypoxia tolerance and its temperature sensitivity can be understood as arising from underlying metabolic O_2_ demand and organismal supply traits (figure 2*c,d*). Active hypoxia tolerance is equivalent to the ratio of organismal O_2_ supply efficacy (*α*_S_) to resting metabolic O_2_ demand rate (*α*_D_), reduced by activity level, *Φ*_crit_: *A*_eco_ = *α*_S_/(*α*_D_*Φ_crit_) (electronic supplementary material) [9]. On their own, observed variations in species resting and active metabolic rates yield a latitudinal pattern of *A*_eco_ similar to diagnosed and measured *A*_eco_, with central values in hypoxia tolerance from metabolic rate alone increasing from high to low latitudes (black circles and triangles in figure 2*c*). This is because species temperature-normalized resting and active metabolic rates are observed to increase significantly, albeit weakly, with absolute latitude (*R*^2^ = 0.09 and 0.15, respectively, *p* < 0.01), such that at the same temperature, tropical species with slower metabolisms would have a lower demand for O_2_ than more metabolically active, high-latitude ones (electronic supplementary material, figure S4). Gradients in organismal O_2_ supply rates versus latitude might also contribute to patterns of *A*_eco_, but there are fewer observations of this trait and latitude trends are weak in available data. The temperature sensitivity of resting metabolic rate (*E*_d_; black circles in figure 2*d*) and O_2_ supply (*E*_s_) also show little apparent trends with latitude but estimates of these parameters are largely missing from the Southern Ocean where the latitude gradient is expected to be strongest based on diagnosed *E*_eco_.

On their own, neither T nor pO_2_ show much skill in explaining the inhabited T-pO_2_ state-space of individual species (figure 1*d,e*; electronic supplementary material, figure S5). Otherwise, mean species temperature sensitivities (*E*_eco_) would centre on 0 (no temperature dependence) or at extreme values (|*E*_o_| > 2), approximating pO_2_-independent temperature thresholds (figure 2*d*). Moreover, spatial variations in diagnosed species *A*_eco_ and *E*_eco_ do not reflect the relationship of environmental pO_2_ versus T in time and depth from local ocean depth profiles, as would be expected if inferred traits were an artifact of the ocean's vertical stratification or of poorly sampled ranges (electronic supplementary material, figure S6).

**Figure 3.**
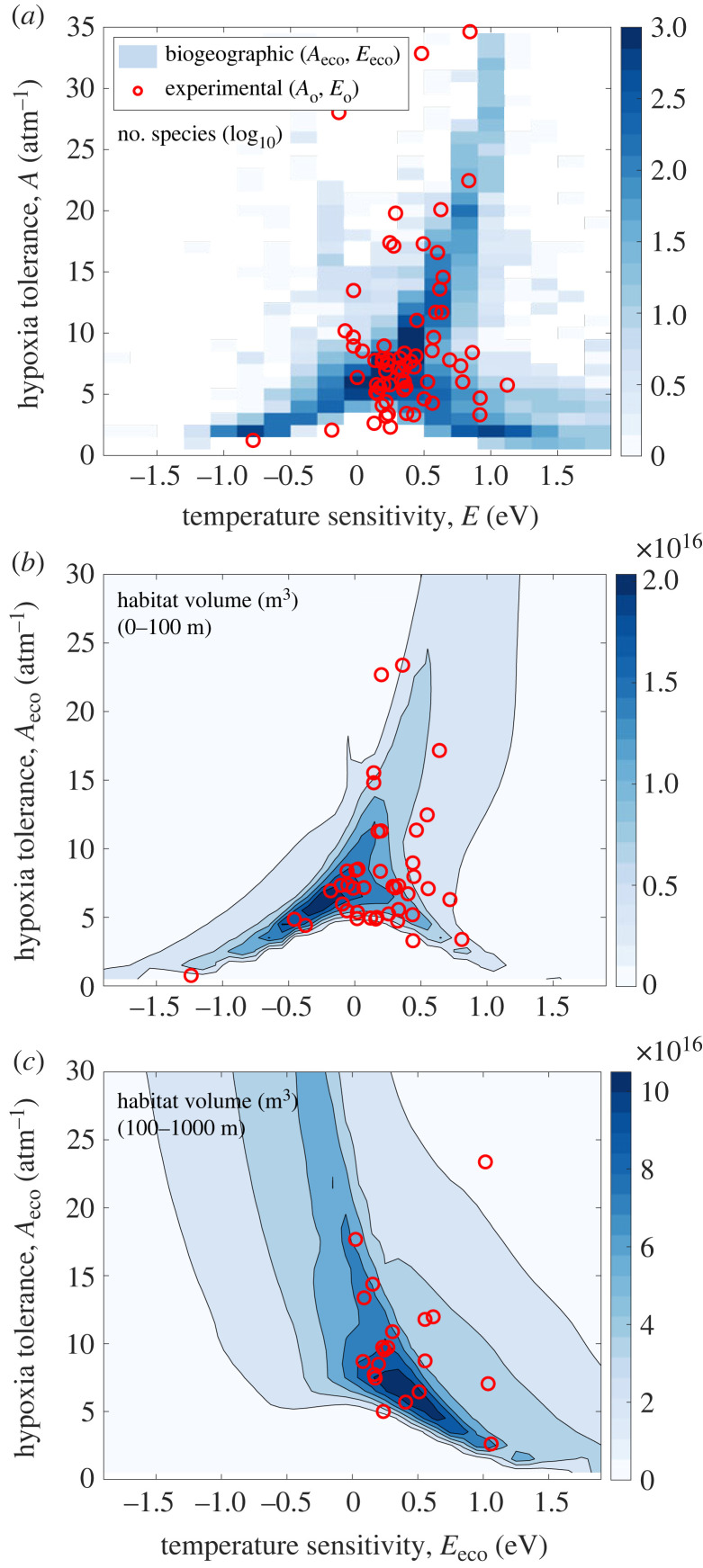
Joint frequency distributions of hypoxia tolerance and its temperature sensitivity compared to habitable ocean volume. (*a*) The joint frequency distributions of active and resting hypoxia tolerance versus their temperature sensitivities are consistent with those expected if trait frequency is influenced by aerobic habitat availability (*b,c*). (*a*) The number of species with a given trait combination of active *A*_eco_ and *E*_eco_ estimated from biogeographic data (colours; log10) is compared to resting traits *A*_o_ and *E*_o_ derived from laboratory experiments (red points). *A*_o_ is normalized by a constant *Φ*_crit_ of 3. There is a positive relationship between *E*_eco_ and *A*_eco_ values with aerobic habitat available in the relatively warm upper 100 m (*b*) but this relationship changes sign in cooler deeper waters (100–1000 m; *c*). Available habitat is defined as the water volume (m^3^) with 1 ≤ *Φ* ≤ *Φ*_max_, where *Φ*_max_ is the median upper *Φ* value inhabited by marine species (figure 4*b*) and assumes a d*E*/d*T* of 0.025 eV/°C  [9]. *E*_eco_ is plotted at species median inhabited temperatures (*a*) and at *T*_ref_ (*b,c*). In (*b,c*), laboratory *A*_o_ is normalized by the species-specific *Φ*_crit_ (data from [9]) and laboratory *E*_o_ is shifted by the median offset from biogeographic *E*_eco_ (red points).

The joint distribution of diagnosed *E*_eco_ and *A*_eco_ reveals new structure in the relationship between active hypoxia tolerance and its temperature sensitivity (figure 3*a*). At low active hypoxia tolerance, inter-species variations in *E*_eco_ increase and display two prominent tails, largely corresponding to tropical (*E*_eco_ < 0), and polar species (*E*_eco_ > 0). At high hypoxia tolerance, most commonly found in tropical to mid-latitudes species, inter-species variations in *E*_eco_ largely converge towards increasingly positive values with larger *A*_eco_. The presence of these distinct tails of the joint distribution of active traits is not readily evident in the relatively sparse experimental data of resting traits alone but is supported by it.

If temperature-dependent O_2_ thresholds constrain the geographical ranges of marine species and thereby influence population fitness, we might expect species trait frequencies to reflect global aerobic habitat availability. We tested the simple hypothesis that traits providing more voluminous aerobic habitat are found among a larger number of species by computing the global aerobic habitat volumes (m^3^) for a given combination of *E*_eco_ and *A*_eco_ using the long-term observations of O_2_ and T (figure 3*b,c*). In the warm, shallow ocean (0–100 m), there is a positive association between the *E*_eco_ and *A*_eco_ with available habitat. Habitat volumes are largely afforded to species with negative *E*_eco_ values and low *A*_eco_ and those with positive *E*_eco_ and high *A*_eco_. By contrast, in colder, deeper waters, the relationship between *E*_eco_ and *A*_eco_ with available habitat changes sign—as *E*_eco_ increases, the *A*_eco_ with available habitat declines. Across depth, this change in slope in the relationship between *E*_eco_ versus *A*_eco_ values with available habitat is consistent with the shape of the diagnosed *E*_eco_ versus *A*_eco_ frequency distribution (figure 3*a*), and with estimates of active traits based on laboratory data (red points in figure 3*b,c*), suggesting global aerobic habitat volume acts as a selective pressure on the global frequency of species traits.

**Figure 4.**
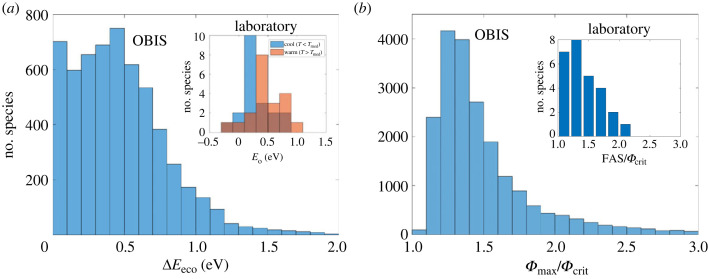
Measures of ‘cold edge’ habitat limits shaped by *Φ*. (*a*) Frequency distribution of the change in species *E*_eco_ (Δ*E*_eco_) from cold to warm habitat limits. Across species, differences in the temperature sensitivity of active hypoxia tolerance (*E*_eco_) over the inhabited *T* range are qualitatively consistent with differences observed from direct laboratory data of resting hypoxia tolerance (*E*_o_) above and below the median experimental temperature (*T*_med_) (inset in (*a*); data from [14]) and predicted in a mechanistic organismal model that includes multi-step O2 supply [25]. The effective T sensitivity of active hypoxia tolerance at the warm (*T*_W_) and cold (*T*_C_) range edges is estimated as *E*_ecoW,C_ = *E*_eco_(*T*_ref_) + $\frac{dE}{dT}$ (*T*_W,C_ − *T*_ref_), where *T*_W_ is the 95th and *T*_C_ is the 5th percentile of inhabited temperatures, and *E*_eco_(*T*_ref_) and $\frac{dE}{dT}$ are species-specific traits. (*b*) Frequency distribution of species maximum occupied *Φ* values (*Φ*_max_) relative to *Φ*_crit_ (95th percentile). The median species inhabits waters with up to 1.4 times as much *Φ* as needed for sustained ecological activity. The ratio of maximum metabolic rate to resting metabolic rate (FAS = MMR : RMR) measured in laboratory experiments is also a factor of ≈40% higher than the *Φ*_crit_ required to sustain a species population in the environment (inset in (*b*); data from [9]).

The inferred trait distributions allow a more comprehensive examination of the role of *Φ* at the cold edge of species geographical ranges. For some species, the lower limit of inhabited pO_2_ increases at cooler temperatures, indicating a shift in the thermal sensitivity of O_2_ supply and thus the potential for hypoxia tolerance to restrict habitat even at cold edges (figure 1*d,e*). We computed the effective T sensitivity of active hypoxia tolerance at warm and cold range edges (figure 4*a*). We find a mean change in effective *E*_eco_ (Δ*E*_eco_) across species temperature ranges of approximately 0.4 eV, from just below 0 in cold water to approximately 0.4 eV at the warm edge, which is about twice the mean difference in resting *E*_o_ in cold versus warm water from laboratory measurements (Δ*E*_o_ = 0.2 eV; inset figure 4*a*) [14], and is owing to the larger $\frac{dE}{dT}$ in diagnosed traits (mean 0.05 ± 0.03 s.d., eV/^o^C) compared to laboratory data ($\frac{dE}{dT}$ ≈ 0.022, 0.013–0.036 range, eV/°C) [9]. A change in *E*_eco_ across temperature is qualitatively consistent with the switch from O_2_ supply limitation via diffusion (*E*_s_ ≈ 0.05 eV) in warm water to ventilation and/or internal circulation (*E*_s_ ≈ 0.4) in cold water [25].

Even for species without a thermal optimum in hypoxia tolerance, *Φ* has the potential to influence habitat at species cold and/or high O_2_ edges via physiological and ecological mechanisms apart from direct aerobic limitation of metabolic demand. We diagnosed the ratio of highest to lowest occupied *Φ* for each species (*Φ*_max_/*Φ*_crit_), which displays a well-defined frequency distribution with a median of approximately 1.4 (1.2 to 2.2, inter-decile range; figure 4*b*), indicating that the typical species inhabits waters with up to 40% higher O_2_ supply than is required for population sustenance. The ratio of maximum metabolic rate to resting metabolic rate (MMR : RMR) measured in laboratory experiments is also a factor of approximately 1.4 higher than the *Φ*_crit_ required to sustain a species population in the environment (inset figure 4*b*) [9,34].

Diagnosed global trait distributions also allow for evolutionary analyses of trait variation across taxa. Taxonomic signals in *A*_eco_ and *E*_eco_ might be expected because related species share similar morphological structures and physiological constraints on metabolism and O_2_ supply, factors that govern hypoxia tolerance [9] and which could be influenced by evolutionary history if species in different groups evolved under distinct climate conditions [35].

**Figure 5.**
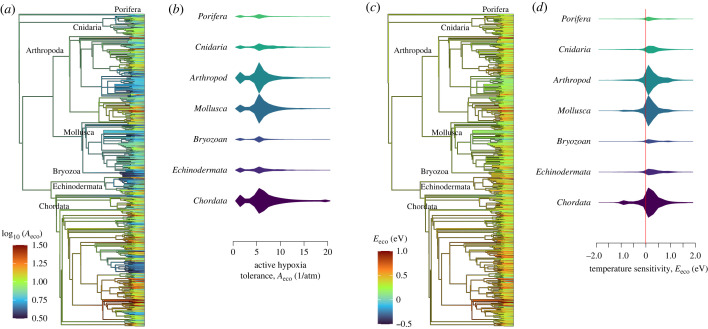
Phylogenetic variation of active temperature-dependent hypoxia traits. Closely related species on the phylogenetic tree can show similar (*a*) active hypoxia tolerances, *A*_eco_, and temperature sensitivities (*c*), *E*_eco_ (colours). Phylogenetic relationships are constructed using the TreeofLife database [37]. In (*a*) and (*c*), tree branch lengths are set equal to the number of descendant tips minus one [38] and use a Grafen's rho parameter, which influences the ratio of branch lengths at tree roots versus tips, equal to 0.4, found to best explain observed variations in resting hypoxia tolerance in fishes [36]. Inter-taxa variations in traits decrease at higher ranks, such as phyla (labelled). (*b,d*) Intra-phyla distributions of *E*_eco_ and *A*_eco_ show convergence across phyla. Trees in (*a,c*) show a sample (1997 species with F1 > 0.8) of the full intra-phyla distributions plotted in kite diagrams (*b,d*), which are limited to phyla with trait estimates available for more than 500 species.

We mapped taxonomic variations in *A*_eco_ and *E*_eco_ across the phylogenetic tree of marine animals using the Open Tree of Life (https://tree.opentreeoflife.org) following methods of [36] (figure 5). We find that both hypoxia tolerance and its temperature sensitivity have a significant phylogenetic signal (Pagel's lambda, *λ* = 0.74 and 0.53, respectively and *p* < 10^−3^), consistent with results for directly measured resting hypoxia tolerance in fishes. This can be observed on the phylogenetic tree, where shared lineages often show similar values of *A*_eco_ and E_eco_. At higher taxonomic ranks towards the tree root, differences between lineages are reduced. Across phyla, distributions of *E*_eco_ and *A*_eco_ show convergence (figure 5*b,d*), indicating that most variation in traits occurs within this higher taxonomic rank, consistent with distributions of resting hypoxia traits [9]. Instead, if taxonomic trait variation primarily occurred at the phylum level, geographical niche differentiation across phyla would be expected. For example, phyla with high active hypoxia tolerances that increase with T would be restricted to warm, low O_2_ conditions while those with lowest tolerances and high *E*_eco_ would only be found in cold waters, in contrast to observed global biodiversity patterns of individual phyla, which have widespread distributions [39]. These observations suggest convergence of species within cosmopolitan taxa, like phyla, on a similar variance in traits across species to be able to inhabit the full breadth of oxythermal conditions present in the ocean. Convergence in diagnosed traits might also arise through ecological interactions which have potential to shape the realized niches of species not directly limited by temperature-dependent hypoxia, but which are tied to species that are so constrained.

**Figure 6.**
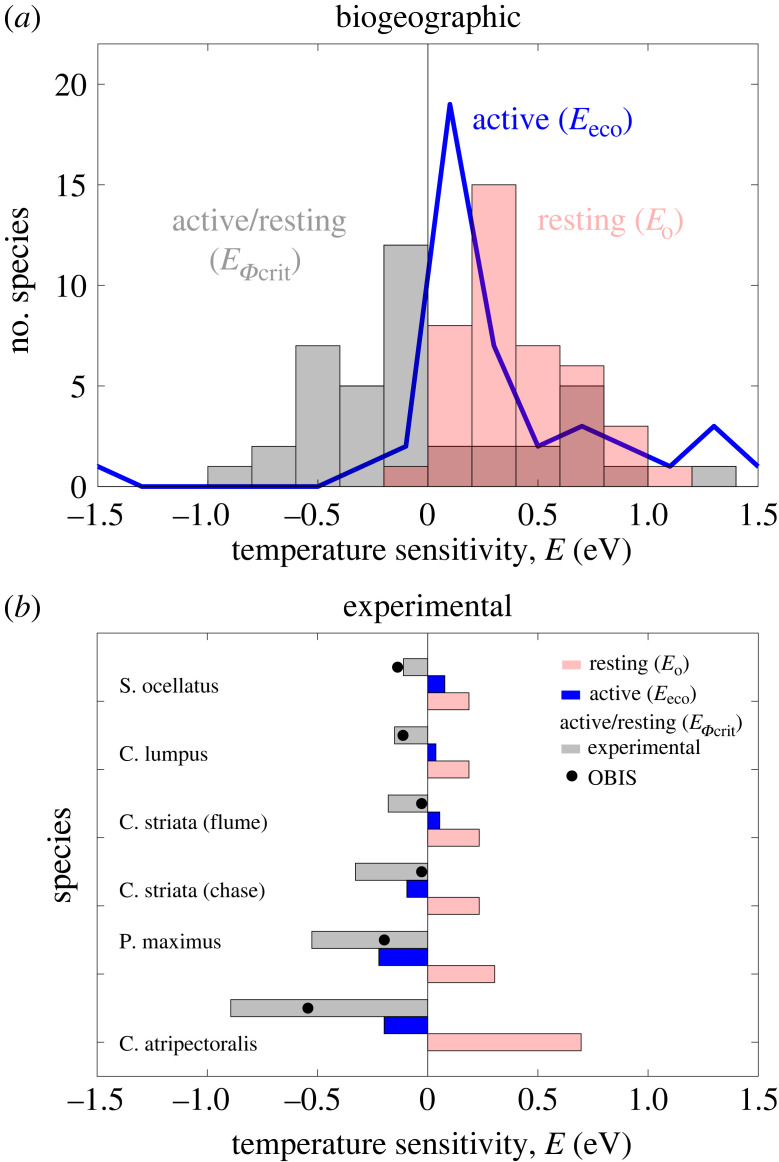
Temperature-sensitivity of active and resting hypoxia tolerance. (*a*) Active hypoxia tolerance varies less with temperature (*E*_eco_) than in the resting state (*E*_o_) because the ratio of active to resting energetic costs (*E_Φ_*_crit_) declines with warming. *E_Φ_*_crit_ distribution in (*a*) is diagnosed from the difference between OBIS (*E*_eco)_ and laboratory (*E*_o_) at the species level. (*b*) Across species, experimental *E_Φ_*_crit_ (grey) derived from ratios of maximum to resting metabolic rates at multiple temperatures (electronic supplementary material) also declines with warming, lowering predicted experimental *E*_eco_ compared measured *E*_o_. *E_Φ_*_crit_ from experimental data (grey bars) and OBIS (points) are correlated at a species-level.

The temperature-dependences of active and resting hypoxia tolerance estimated from biogeographic and laboratory data can be paired to derive a new trait, the temperature dependence (*E_Φ_*_crit_) of the ratio of active to resting metabolic rate (*Φ*_crit_). The value of *Φ*_crit_ has previously been assumed to be independent of temperature [4,9], such that *E_Φ_*_crit_ = 0. Across species, the median *E*_eco_ (0.19 eV) falls below resting *E*_o_ (0.34 eV) (electronic supplementary material, figure S7), suggesting that the ratio of active to resting metabolic rates declines with warming (i.e. *E_Φ_*_crit_ < 0) since *E*_eco_ = *E*_o_ + *E_Φ_*_crit_ (figure 1*b,c*). We estimated the distribution of *E_Φ_*_crit_ at the species level and found that its distribution is indeed shifted below *E*_o_ and has a median value of −0.14 eV (figure 6*a*). Since resting metabolic rates increase with temperature, this implies that for the typical species, active metabolic costs are relatively constant.

We tested this prediction with laboratory measurements of MMR and RMR at multiple temperatures for six species using the definition for FAS (FAS = MMR/RMR), which measures the ratio of MMR to RMR, as a proxy for *Φ*_crit_. This comparison is necessarily approximate because sustained activity in nature falls between MMR and RMR, and includes biological processes not resolved in short term MMR experiments, such as reproduction, feeding, growth and predation, in addition to uncertainty in laboratory measurements. Nevertheless, it serves as an initial check on the expected lower temperature-dependence of active hypoxia tolerance (*E*_eco_) compared to the resting state (*E*_o_). We find that experimental *E_Φ_*_crit_ is consistently below 0 for all species (median *E_Φ_*_crit_ = −0.25 eV) (figure 6*b*), shifting the predicted estimates of *E*_eco_ for these species below their measured *E*_o_. This finding implies that the ratio of active to resting metabolic rate declines with warming as predicted. Moreover, *E_Φ_*_crit_ diagnosed from biogeographic data and estimated from laboratory FAS are correlated at the species level (*R*^2^ = 0.77, *p* = 0.02), although the sample size is small. These findings are also in qualitative agreement with published inter-specific estimates of the temperature-dependence of MMR and resting metabolic rate in fishes [34]. Thus, experimental data on rates of active to resting metabolic rate support the lower temperature-dependence of active compared to resting costs inferred here.

## 4. Discussion

Diagnosing species ecophysiological traits from biogeographic observations allows the active hypoxia tolerance and its temperature sensitivity to be estimated for tens of thousands of well-documented marine species in major animal phyla. Global spatial gradients in species traits appear to reflect adaptations needed to provide metabolically viable habitat under distinct oxythermal conditions, such as elevated active hypoxia tolerances that decline less with temperature in the warm, low O_2_ tropics and highly positive *E*_eco_ that provides habitat in cold waters of the Southern Ocean.

Species range boundaries in T-pO_2_ state-space are consistent with measured temperature-dependent hypoxia thresholds whose mean temperature dependence (*E*_eco_ ≈ 0.2 = *E*_d_ – *E*_s_ + *E_Φ_*_crit)_ can be explained by the temperature sensitivity of organismal O_2_ supply (*E*_s_ ≈ 0.4), and metabolic demand (*E*_d_ ≈ 0.7) [9,25], lowered by the ratio of active to resting metabolic rates (*E_Φ_*_crit_ ≈ −0.1). In support of this mechanistic association, experimental data implies a lower temperature dependence for the cost of activity compared to maintenance, which imparts a decline in *Φ*_crit_ with warming. In addition, changes in diagnosed *E*_eco_ across the oceanic temperature range are consistent with direct laboratory measurements and predictions from an organismal model that includes the effect of changing O_2_ supply mechanisms (figures 1*d,e* and 4). Together, these results support aerobic habitat limitation as a constraint on marine ectotherm species geographical distributions and their sensitivities to climate change.

The correspondence between *Φ*_max_/*Φ*_crit_ and maximum to sustained metabolic rates suggests that O_2_ supply capacity evolves to satisfy maximum metabolic demand [40]. Species occupying waters with O_2_ supply above their maximum metabolic capacity for O_2_ consumption might eventually be excluded via competition for limiting resources, including food, by other species with lower hypoxia tolerance that instead invest available energy elsewhere besides O_2_ supply physiology (e.g. structures to increase gill surface area, ventilation or circulation rates). The large energetic costs of osmoregulation might also limit the benefits of investing in O_2_ supply traits [41]. In addition, parasitic infection rates have been observed to increase with O_2_ ventilation of the gills [42], one of the factors influencing hypoxia tolerance, suggesting species with lower *A*_eco_ (and thus *Φ*_max_) might also have a lower infection risk.

Aerobic habitat constraints also appear to apply across diverse animal phyla, which have converged on a similar average and variance in species traits governing hypoxia tolerance and its temperature sensitivity despite widely varying morphology and physiology. This trait convergence points to a selective pressure to colonize the wide breadth of aerobic conditions found across the ocean. While we cannot rule out convergence in realized niches owing to species ecological interactions, as opposed to fundamental niches owing to ecophysiological traits, one would not expect to find biogeographic niches consistent with temperature-dependent lower pO_2_ curves unless some species were dependent on others, which were fundamentally constrained by the ecophysiological traits in the first place. Moreover, a similar convergence in inter-phyla trait distributions have been found for resting hypoxia tolerance and its temperature sensitivity from laboratory measurements, which include no effect from ecological interactions [9] and are a component of the active traits diagnosed here. The correspondence between diagnosed trait frequencies and global habitat availability suggests that traits providing larger habitats are selected for in a larger number of species. Open questions remain about the mechanistic factors shaping this selection, including how observed central tendencies, variability and trait coexistence is maintained across species, and how trait adaptation might have varied under different climate states in Earth history, with implications for past ecosystem sensitivities to climate change and taxonomic selectivity during marine extinctions [43].

Diagnosing species trait distributions provides a useful constraint on modern species habitat vulnerabilities to anthropogenic climate change. The spatial patterns of species traits diagnosed from biogeographic data support modelled trait gradients for the modern ocean and past time-intervals, which govern extinction patterns in future projections and palaeo simulations [21,23]. Diagnosed trait gradients thereby strengthen the hypothesis that extinction risk from climate warming increases with latitude, as species least hypoxia tolerant (lowest *A*_eco_) and most temperature sensitive (highest *E*_eco_) are found polewards, and have nowhere to seek refuge in a warmer, less oxygenated ocean. By contrast, species from lower latitudes that are more tolerant to hypoxia and with weaker temperature sensitivities might be able to track aerobic habitat niches to higher latitudes as these environments become more tropical, consistent with recently observed species poleward migrations under warming [44].

Diagnosed trait distributions also reveal the current limitations of direct experimental data compilations. Species found at the extremes of the ocean's oxythermal conditions are poorly sampled in laboratory data compared to their oceanic diversity, including for lowest and highest *A*_eco_ and *E*_eco_. Trait estimates from species spanning Southern Ocean latitudes, where strong trait gradients are inferred and prior work indicates O_2_ limitation [45], could provide a valuable test of these findings. The under-sampling of extreme traits in laboratory compilations is unlikely to impact global projections of species vulnerability to climate change [21], but could bias regional assessments. Polar oceans are projected to experience the strongest O_2_ declines under anthropogenic climate change [46], while in the tropics, the frequency of metabolic storms, where hypoxic conditions co-occur with heat waves, is expected to become more severe [20]. Future efforts to understand climate impacts on aerobic habitat viability in the ocean should therefore prioritize species endemic to these biologically unique and climatically vulnerable ecosystems.

## Data Availability

Data, MATLAB and R code are available from the GitHub repository: https://github.com/jlpenn/MI_traits_obis [47]. Supplementary methods, figures, and tables are provided in the electronic supplementary material [48].
